# The tumor suppressive role of CAMK2N1 in castration-resistant prostate cancer

**DOI:** 10.18632/oncotarget.1968

**Published:** 2014-05-13

**Authors:** Tao Wang, Zhuo Liu, Shuiming Guo, Licheng Wu, Mingchao Li, Jun Yang, Ruibao Chen, Hua Xu, Shaoxin Cai, Hui Chen, Weiyong Li, Liang Wang, Zhiquan Hu, Qianyuan Zhuang, Shaohua Xu, Liping Wang, Jihong Liu, Zhangqun Ye, Jun-Yuan Ji, Chenguang Wang, Ke Chen

**Affiliations:** ^1^ Department of Urology, Tongji Hospital, Tongji Medical College, Huazhong University of Science and Technology, Hubei, China; ^2^ Institute of Urology, Tongji Hospital, Tongji Medical College, Huazhong University of Science and Technology, Hubei, China; ^3^ Department of Surgery, Tongji Hospital, Tongji Medical College, Huazhong University of Science and Technology, Hubei, China; ^4^ Department of Radiology, Tongji Hospital, Tongji Medical College, Huazhong University of Science and Technology, Hubei, China; ^5^ Union Hospital, Tongji Medical College, Huazhong University of Science and Technology, Hubei, China; ^6^ Kimmel Cancer Center, Department of Cancer Biology, Thomas Jefferson University, Philadelphia, PA, USA; ^7^ Department of Molecular and Cellular Medicine, College of Medicine, Texas A&M University Health Science Center, College Station, TX 77843, USA

**Keywords:** CAMK2N1, prostate cancer, tumor suppressor

## Abstract

Prostate cancer at advanced stages including metastatic and castration-resistant cancer remains incurable due to the lack of effective therapies. The CAMK2N1 gene, cloned and characterized as an inhibitor of CaMKII (calcium/calmodulin-dependent protein kinase II), has been shown to affect tumorigenesis and tumor growth. However, it is still unknown whether CAMK2N1 plays a role in prostate cancer development. We first examined the protein and mRNA levels of CAMK2N1 and observed a significant decrease in human prostate cancers comparing to normal prostate tissues. Re-expression of CAMK2N1 in prostate cancer cells reduced cellular proliferation, arrested cells in G_0_/G_1_ phases, and induced apoptotic cell death accompanied by down-regulation of IGF-1, ErbB2, and VEGF downstream kinases PI_3_K/AKT, as well as the MEK/ERK-mediated signaling pathways. Conversely, knockdown of CAMK2N1 had a significant opposite effects on these phenotypes. Our analyses suggest that CAMK2N1 plays a tumor suppressive role in prostate cancer cells. Reduced CAMK2N1 expression correlates to human prostate cancer progression and predicts poor clinical outcome, indicating that CAMK2N1 may serve as a biomarker. The inhibition of tumor growth by expressing CAMK2N1 established a role of CAMK2N1 as a therapeutic target.

## INTRODUCTION

Prostate cancer is one of the most common malignancies among men and the second most common cause of male cancer-related deaths [1]. The usual progression of prostate cancer goes from castration-sensitive to -resistant, inevitably developing highly metastatic properties [2]. Prostate tumors initially respond to hormonal intervention therapies, however, with androgen-independence emerging, tumors develop resistance to anti-androgen therapies [3]. The frontline treatments for advanced prostate cancer consist of hormone therapy, chemotherapy, and radiation. Unfortunately, limited therapy options are stalling the survival rates in patients [4, 5].

CAMK2N1 (calcium/calmodulin-dependent protein kinase II inhibitor 1, also known as PRO1489), a peptide composed of 78-amino acids, was initially shown to localize in the cell junction and synapse [6, 7, 8]. CAMK2N1 functions as a potent and specific inhibitor of CaMKII (calcium/calmodulin-dependent protein kinase II). CaMKII is a member of the Calcium/calmodulin (CaM) 2-dependent protein kinase family, and is a ubiquitous serine/threonine protein kinase that phosphorylates nearly 40 different proteins, including enzymes, ion channels, kinases and transcription factors [7, 8]. CaMKII signaling plays a role in cell-cycle progression by activating MEK/ERK to enhance the phosphorylation of p27^Kip1^[8]. Inhibition of CaMKII activity by synthetic agents has been shown to suppress cell growth [9]. In addition, CAMK2N1-mediated inhibition of CaMKII activity regulates the cell-cycle progression in colon cancer cells through de-activation of MEK/ERK kinase activity and p27 protein accumulation [7, 8]. Currently, it is still unknown whether CAMK2N1 plays any role in prostate cancer development.

In this study, we performed immunohistochemical (IHC) staining using human prostate cancer specimens and observed a significant reduction of CAMK2N1 in prostate cancer compared to normal tissue. Decreased CAMK2N1 expression correlated with more advanced stages of prostate cancer. We observed that overexpression of CAMK2N1 significantly impaired human prostate cancer cell proliferation and tumor growth *in vivo*, while depletion of CAMK2N1 promoted cell proliferation and tumor growth. Genome-wide gene profiling revealed that CAMK2N1 regulated the expression of key proteins associated with cell-cycle progression and apoptosis. Taken together, our findings revealed a tumor suppressive role for CAMK2N1 and established CAMK2N1 as molecular determinant in hormone sensitivity of prostate cancer.

## RESULTS

### Reduced CAMK2N1 expression in human prostate cancer

**Figure 1:**
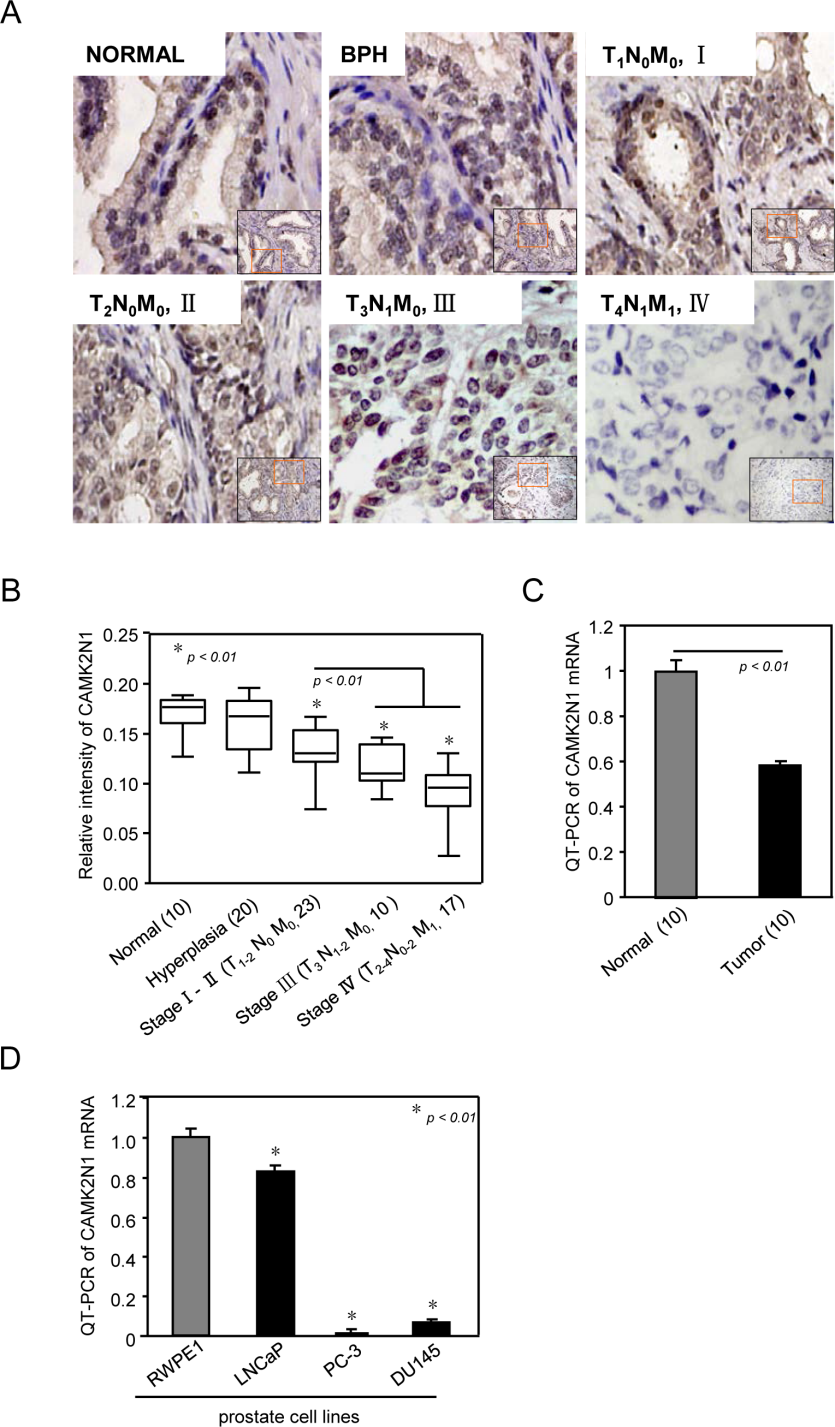
CAMK2N1 abundance is reduced in human prostate cancer (A) Representative examples of immunohistochemical staining for CAMK2N1 in each of the clinical stages of prostate cancers as indicated. (B) Quantification of CAMK2N1 relative immunostaining intensity for each clinical stage of prostate cancers. Data is shown as mean ± SEM for N as indicated in the figure in parethesis as shown. (C) CAMK2N1 mRNA determined by quantitative PCR. Comparison was made between normal and tumorous prostate samples. (D) QT-PCR analysis of CAMK2N1 expression in prostate cell lines (RWPE1, LNCaP, DU145, PC3).

We first analyzed CAMK2N1 protein expression in human prostate cancer specimens by performing immunohistochemical (IHC) staining. In normal prostate tissue, CAMK2N1 showed whole cell distribution with stronger staining in the nucleus compared to cytoplasm (Fig. 1A). Hyperplastic and normal tissues had similar expression levels and distribution patterns (Fig 1A-B). However, CAMK2N1 protein levels were decreased in cancerous tissues with moderate expression in early stages (I­/II) and poor expression in advanced stages (III/IV). The nuclear CAMK2N1 staining was quantified in human prostate tumors (n = 50), hyperplastic tissues (n = 20), and normal control specimens (n = 10) (Fig. 1B). Next, we analyzed the abundance of CAMK2N1 mRNA in prostate cancer tissues compared to matched normal-like control from same patient (n = 10) by quantitative RT-PCR (qRT-PCR) analysis. As shown in Fig. 1C, the mRNA level of CAMK2N1 was significantly reduced in prostate cancer tissues compared to normal prostate, which is consistent with the reduced protein expression in prostate cancer cells.

Next, we examined CAMK2N1 mRNA levels by qRT-PCR in several most commonly used prostate epithelial cells, including AR-negative PC3 and DU145 cells, AR-positive castration-sensitive LNCaP cell and RWPE1, a normal prostate cell line serving as control. As shown in Fig. 1D, all prostate cancer cells exhibited decreased expression of CAMK2N1, with LNCaP being closest to the control. Taken together, these observations suggest that the level of CAMK2N1 is reduced in both human prostate cancer patient samples and commonly used prostate cancer cells.

### CAMK2N1 inhibits prostate cancer cell proliferation and cell cycle progression

**Figure 2:**
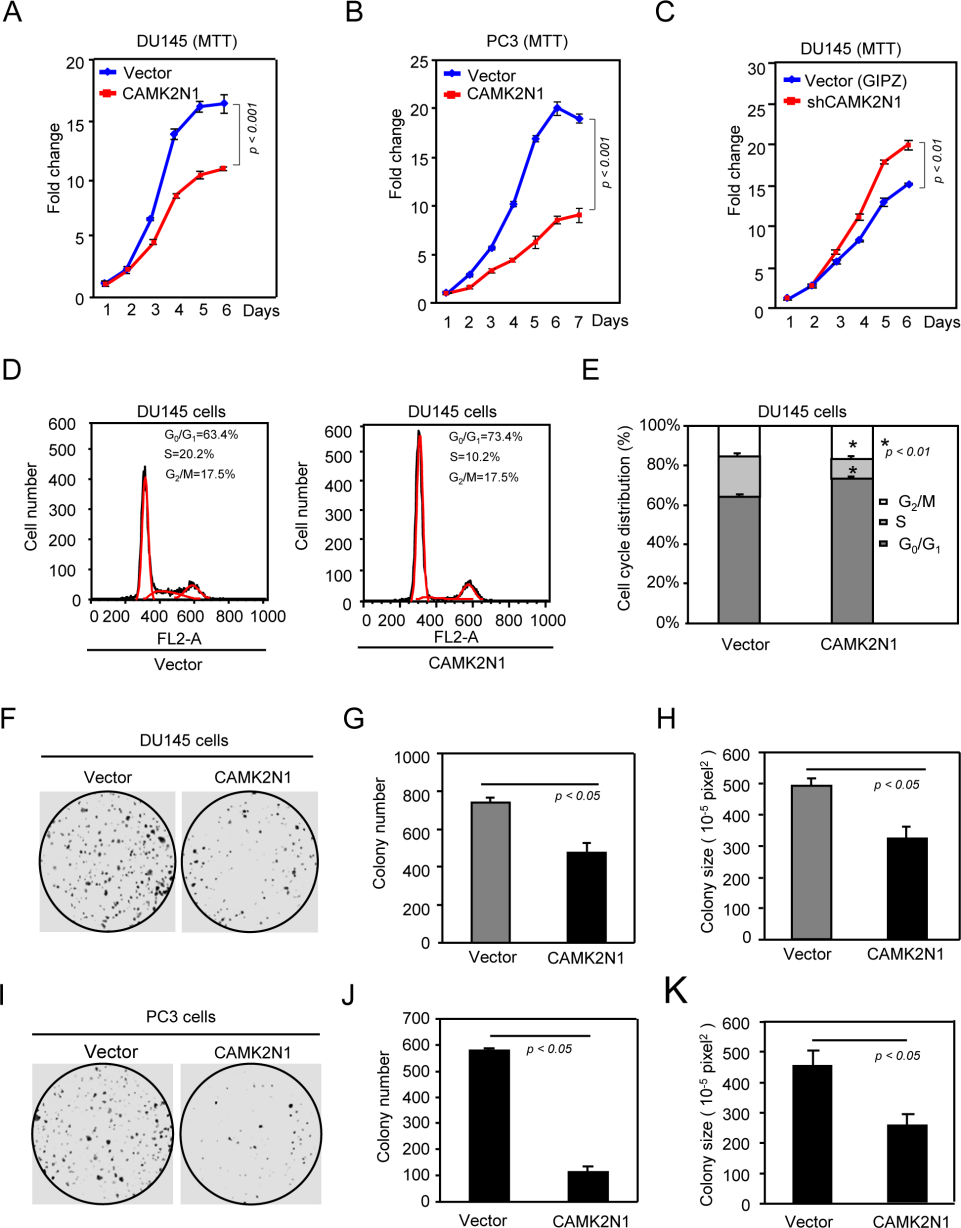
CAMK2N1 inhibits prostate cancer cell proliferation and cell cycle progression DU145 and PC3 cells stably overexpressed or knocked down CAMK2N1. Cells were analyzed for cell proliferation by (A-C) MTT, cell cycle by (D-E) FACS and oncogenic growth by (F-K) Colony-forming assay. Data is shown as mean ± SEM for N > 5 separate experiments.

To determine the functional significance of CAMK2N1 in regulating human prostate cancer growth, we first analyzed whether CAMK2N1 regulates the proliferation of human prostate cancer cells by MTT assays. We found that overexpression of CAMK2N1 in DU145 and PC3 cells inhibited cellular proliferation (Fig. 2A-B), and conversely, depletion of CAMK2N1 in DU145 cells using shRNAs targeting CAMPK2N1 enhanced cell proliferation (Fig. 2C). To further analyze the effect of CAMK2N1 on cell-cycle progression, FACS analysis was conducted. Overexpression of CAMK2N1 increased G_0_/G_1_ cell fraction and reduced the proportion of cells in S-phase after 24 hrs post serum induction in serum-starved DU145 and PC3 cells (Fig. 2D-E, Supplemental Fig. 2A), while knockdown of CAMK2N1 in DU145 cells with shRNA reduced the proportion of cells in G_0_/G_1_ and increased the proportion of cells in S-phase (Supplemental Fig. 1A).

To further determine whether CAMK2N1 inhibits oncogenic growth of prostate cancer cells, we performed colony formation assays. Ectopic expression of CAMK2N1 inhibited both the number and the size of colonies by 30% - 40% (Fig. 2F-K,), while the shRNA-mediated knockdown of CAMK2N1 enhanced DU145 colony formation (Supplemental Fig. 1B).

### CAMK2N1 inhibits prostate tumor growth *in vivo*

**Figure 3:**
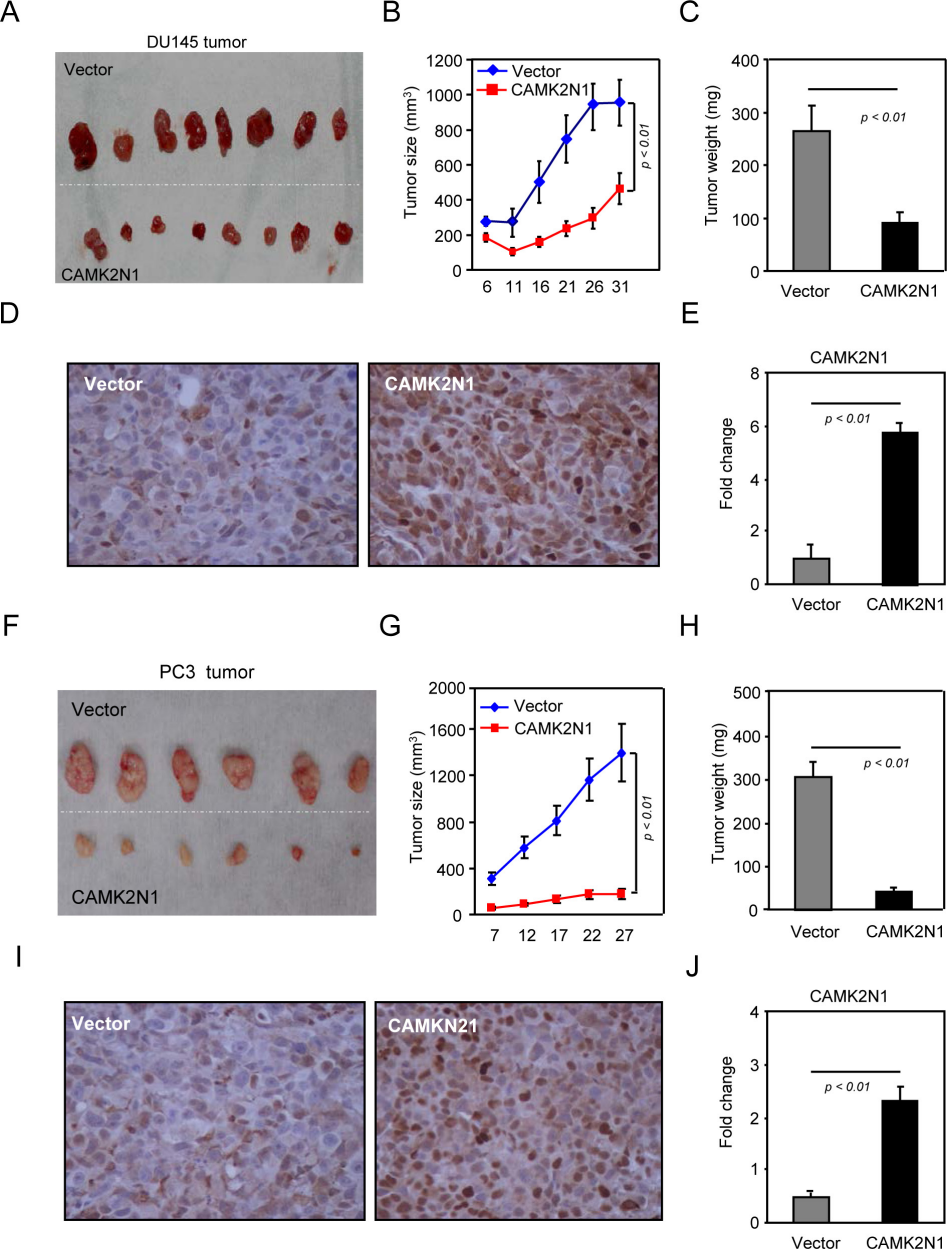
CAMK2N1 inhibits prostate tumor growth *in vivo* (A-J) Serial measurements were conducted every five days of DU145 and PC3 tumors stably expressing CAMK2N1, injected into nude mice. The data is shown as mean ± SEM for N>6 separate tumors for each group. (A, F) Images of tumors dissected out from the sacrificed mice. (B, G) The tumor size (mm3) versus days of post injection. (C, H) Reduction in tumor weight after resection at the end of experiment. (D-E, I-J) IHC staining detected the expression of CAMK2N1 in DU145 and PC3 tumor tissues of nude mice.

Encouraged by these observations, we then investigated the role of CAMK2N1 in inhibiting prostate tumor growth *in vivo*. DU145 and PC3 cells stably transduced with an expression vector encoding CAMK2N1 were implanted subcutaneously into immune-deficient mice. Tumor size was measured every 5 days with last measuring performed on day 32 (Fig. 3A, F). We observed that that overexpression of CAMK2N1 significantly reduced both the tumor size (Fig. 3B,G) and the tumor weight (Fig. 3C, H). IHC staining demonstrated the expression of CAMK2N1 (Fig. 3D, E, I, J). These observations suggest that CAMK2N1 negatively regulate prostate tumor growth *in vivo*.

### CAMK2N1 induces apoptotic cell death in human prostate cancer cells

**Figure 4:**
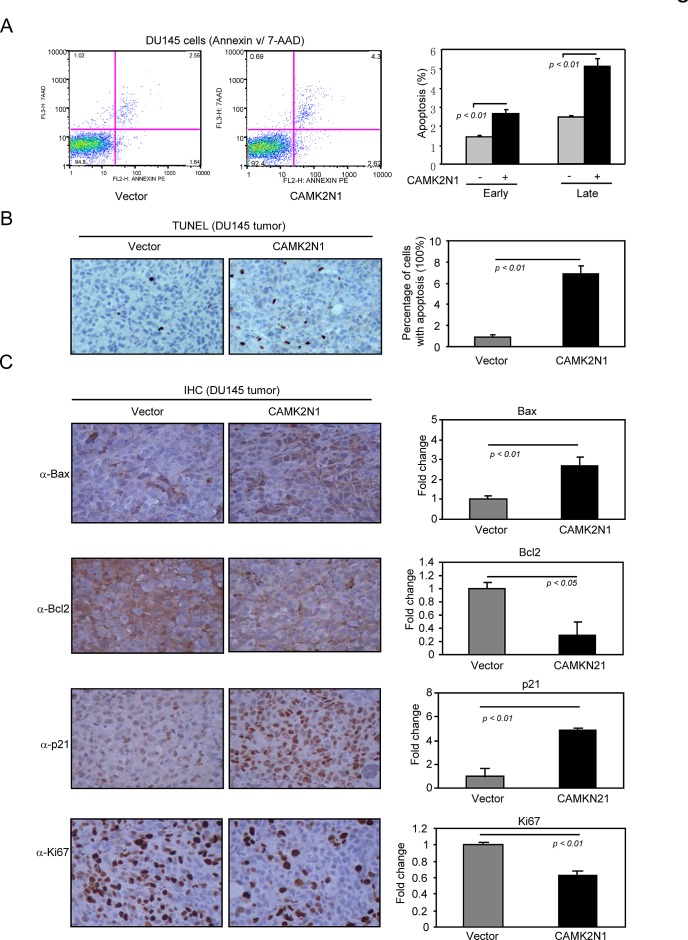
CAMK2N1 induces apoptotic cell death in human prostate cancer cells DU145 cells stably overexpressed CAMK2N1. These cells were analyzed for apoptosis by (A) Annexin V staining. Quantification of colony numbers and sizes were shown as mean ± SEM for N>5 separate experiments. (B) TUNEL staining was conducted to assess the effect of CAMK2N1 on cellular apoptosis *in vivo*. The percentage of apoptotic cells was increased in DU145 tumor tissues derived from nude mice. Quantification of TUNEL staining was shown as mean ± SEM for N=4 separate experiments. (C) IHC staining detected the expression of Bax, Bcl2, p21 and Ki67 in DU145 tumor tissue of nude mice. Overexpression of CAM2KN1 in DU145 and PC3 tumor tissue decreased Bcl-2, Ki67 protein expression and increased p21, Bax protein expression. Data for quantified IHC was shown as mean ± SEM for N = 4 tumors in each group.

Since elevated cell death may also contribute to impaired tumor growth, we determined whether CAMK2N1 induces apoptosis by Annexin V staining and TUNEL assays. Annexin V staining revealed that proportion of cells in early and late apoptosis was increased in CAMK2N1 overexpressing DU145 cells (Fig. 4A) and PC3 cells (Supplemental Figure 2B). TUNEL staining was conducted to assess the effect of CAMK2N1 on cell apoptosis in tumor samples from *in vivo* experiment. Similarly, the percentage of apoptotic cells was increased in CAMK2N1 overexpressing tumor derived from nude mice implanted with DU145 cells (Fig. 4B). To determine the expression of Bax, Bcl-2, p21, and Ki67 in tumor tissues, we conducted IHC staining in tumors with over-expressed CAM2KN1. As shown in Fig. 4C, tumors overexpressing CAM2KN1 have reduced expression of Bcl-2, Ki67 and increased p21 and Bax expression, suggesting that gain of CAM2KN1 promotes apoptosis in prostate cancer cells.

### CAMK2N1 suppresses IGF-1, ErbB2, VEGF, downstream kinase-PI_3_K/AKT, MEK/ERK, and HSP27-mediated signaling pathways in prostate cancer cells

**Figure 5:**
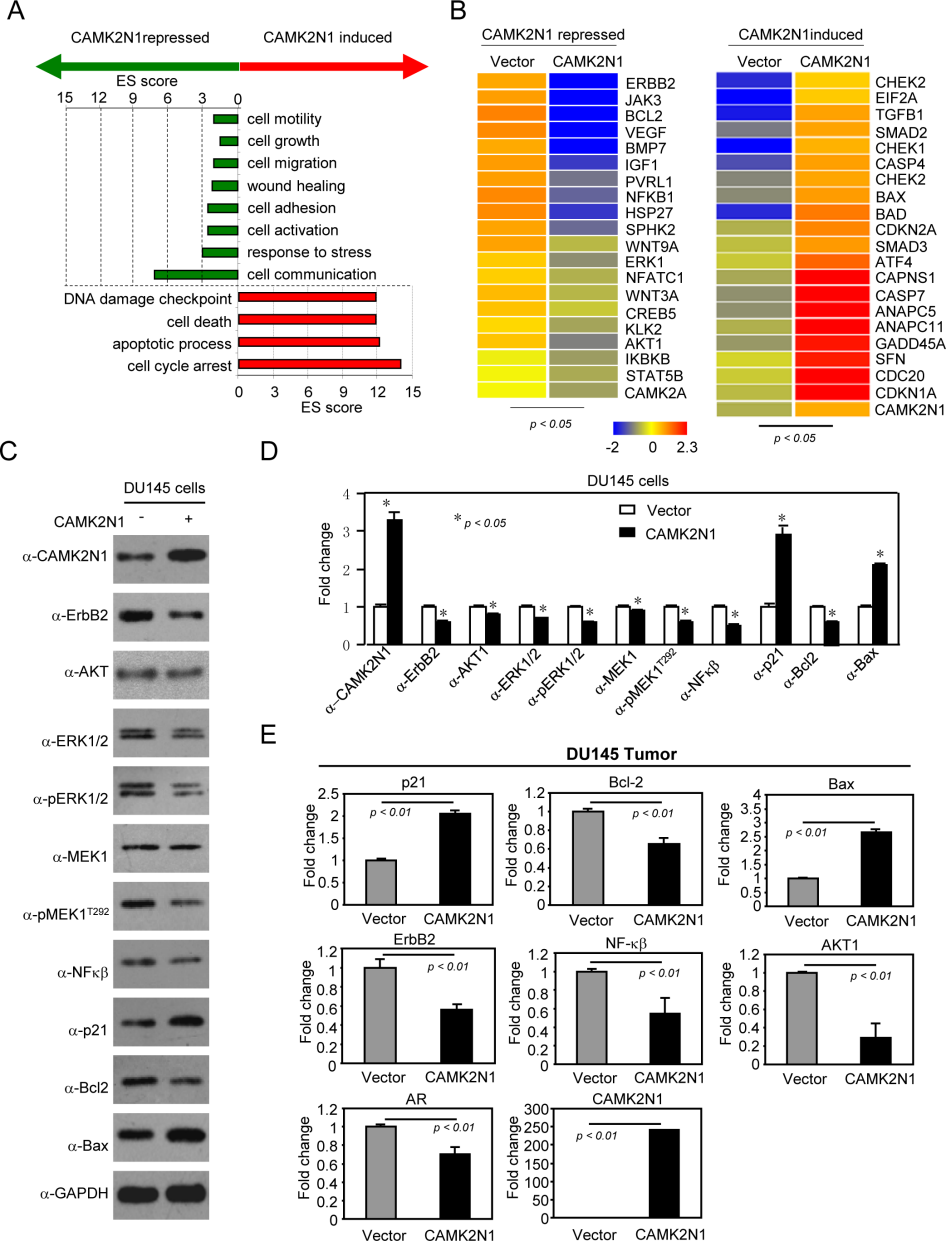
CAMK2N1 suppresses IGF-1, ErbB2, VEGF, downstream kinase-PI_3_K/AKT, MEK/ERK and HSP27-mediated signaling pathways in prostate cancer cells (A-B) Microarray gene expression analysis was conducted in DU145 cells stably overexpressing CAMK2N1 (>1.5-fold and *p* < 0.05). (A) Functional analysis of differentially expressed genes. Gene Ontology (GO) Biological Process (BP) terms were ranked by score (score > 1.3). (B) CAMK2N1 induced and repressed clusters of genes that were chosen from the pathways. (C-D) Expression levels of ErbB2, AKT, MEK1, ERK1/2, NFκβ, Bcl-2, BAX, and p21 were determined by Western blot in DU145 cells with stably overexpressing CAMK2N1. Similar changes as to those found in the microarray analysis were observed. Each figure represents three independent experiments. (E) DU145 tumors stably expressing CAMK2N1, and mRNA levels of ErbB2, Bcl-2, NF-κβ, AKT1, AR, p21, and Bax were determined by qRT-PCR. CAMK2N1 decreased ErbB2, BCL-2, NF-κβ, AKT1, AR mRNA levels and increased p21, Bax mRNA levels in DU145 tumor tissues. The data is shown as mean ± SEM for N=4 separate tumors for each group.

To determine the mechanisms by which CAMK2N1 functions to inhibit prostate tumor growth, we performed genome-wide gene expression analysis using human 12×135K gene expression array by comparing DU145 cells with or without CAMK2N1 overexpression (> 1.5-fold and *p* < 0.05). Pathway analysis conducted on genes differentially regulated by CAMK2N1 revealed several up-regulated signaling pathways, including pathways that regulate cell-cycle arrest, apoptosis, and DNA damage responses (Fig. 5A). This analysis also identified several down-regulated pathways, including pathways that control cell growth, cell migration, cell adhesion, stress response, blood vessel development, and cell communication. CAMK2N1 induced the expression of genes encoding CDKN1A, BAX, BAD, GADD45A, SFN, CAPNS1, CHEK1, CHEK2, CDKN2A, ANAPC11, ANAPC5, SMAD2, SMAD3, CDC20, TGFB1, CASP4, CASP7, ATF4, and EIF2A, while CAMK2N1 repressed the expression of genes including IGF1, AKT1, BCL2, VEGF, ERK1, IKBKB, NFKB1, STAT5B, CREB5, ERBB2, BMP7, HSP27, CAMK2A, JAK3, PVRL1, WNT3A, WNT9A, NFATC1, KLK2, and SPHK2 clusters of genes (Fig. 5B). KEGG (Kyoto Encyclopedia of Genes and Genomes) analysis identified an interconnected network, including five major kinase pathways: IGF1, VEGF, ERBB2, MEK/ERK, PI3K/AKT, which are involved in cell growth, survival, migration or invasion, and angiogenesis (Supplemental Figure 3A).

To validate the microarray data, we performed Western blot and qRT-PCR analyses and focused on a subset of genes that are either down-regulated (ErbB2, AKT, MEK1, ERK1/2, Bcl-2) or up-regulated (p21, Bax). Overexpression of CAM2KN1 in DU145 and PC3 cells resulted in decreased ErbB2, MEK1, pMEK1^T292^, ERK1/2, p-ERK1/2, AKT, pAKT^ser473^, Bcl-2, NF-κB protein expression and increased p21 and Bax protein expression (Fig. 5C-D, Supplemental Figure 2C). Overexpression of CAM2KN1 in DU145 tumor tissues resulted in decreased ErbB2, AKT1, Bcl-2, NF-κB and AR mRNA levels and increased p21, Bax mRNA levels (Figure 5E). Conversely, knockdown of CAMK2N1 by shRNA in DU145 cells led to an opposite effect on protein expression of these genes (Supplemental Figure 1C). Taken together, these data showed that CAMK2N1 regulates cell proliferation, apoptosis and tumor growth *in vivo*, likely through the functional interactions with these signaling molecules in prostate cancer cells.

## DISSCUSSION

In this study, we demonstrated that CAMK2N1 plays a tumor suppressive role in human prostate cancer. CAMK2N1 is significantly reduced in prostate cancer compared to benign and normal prostate tissues. Reduced CAMK2N1 expression positively correlated with prostate tumor progression (stage, TNM). Consistent with the expression pattern in normal and malignant prostate tissues, CAMK2N1 was expressed at a higher level in non-tumorigenic prostate epithelial cells. In addition, a moderate expression of CAMK2N1 was detected in castration sensitive LNCaP cells, while its expression was relatively low in castration-resistant prostate cancer DU145 and PC3 cells. Overexpression of CAMK2N1 in DU145 and PC3 cells led to the inhibition of cell proliferation, accumulation in G_0_/G_1_ phase and the induction of apoptosis, while CAMK2N1 knockdown promoted S-phase entry after release from G_0_/G_1_ synchronization and enhanced cell proliferation in DU145 cells.

CAMK2N1 is the specific inhibitor of CaMKII. As a ubiquitous serine/threonine protein kinase, CaMKII phosphorylates nearly forty different proteins, including enzymes, ion channels, kinases and transcription factors [7, 8]. In previous studies, CAMKII was involved in insulin-induced cell proliferation [10]. CaMKII phosphorylates Raf-1 at S338 and participates in ERK activation upon different stimuli [11]. Furthermore, CAMK2N1-mediated inhibition of CAMKII activity caused deactivation of MEK/ERK kinase activity and accumulation of p27 protein, which primarily regulates the cell cycle progression of colon cancer cells [7, 8], suggesting a role of CAMK2N1 in tumorigenesis. However, the role of CAMK2N1 in cancer has not been investigated in more details. PI3K/AKT and MEK/ERK signaling pathways have been shown to cooperate in prostate cancer progression and the transition to castration resistant disease [12]. Inhibition of these signaling pathways acts combinatory to suppress the pathway activation and inhibit tumor growth, cellular proliferation and migration in prostate cancer [13]. Analyses of human prostate tumor specimens suggest that PI3K/AKT and MEK/ERK signaling pathways are frequently activated by IGF-1, ErbB2, and VEGF in prostate tumors [13, 14, 15], via the phosphorylation, subsequent activation of NF-κB, Bcl2 protein, and deactivation of p21 and Bad protein. Increased activation of the PI3K-Akt-mTORC1 pathway is a common aberration in prostate cancer [16]. IGF-1 acts as an important regulator of growth, survival, and metastatic potential in a variety of malignancies and has been shown to be involved in prostatic carcinogenesis and CRPC [17]. IGF-1 also rapidly potentiates the activation of Ca^2+^/CAMKII channel [18]. In this study, we observed that CAMK2N1 suppressed the IGF-1, ErbB2, VEGF expression and their downstream kinase-PI3K/AKT, and MEK/ERK-mediated signaling pathways, which were critical for cell proliferation and survival. Furthermore, CAMK2N1 induced cell cycle regulatory kinases, p21, 14-3-3, Chk1, Chk2, possibly accounting for the G_0_/G_1_ arrest. CAMK2N1 also induced apoptosis regulatory kinases including Bax/Bcl2, Bad, caspase4, caspase7, explaining how CAMK2N1 enhanced cell apoptosis. Collectively, results from our study revealed the tumor suppressive role of CAMK2N1 in prostate cancer.

In summary, we demonstrated that CAMK2N1 expression was reduced in prostate cancer. Increased CAMK2N1 expression suppressed cell proliferation, survival and tumor growth *in vivo*. These data suggested that CAMK2N1 plays an important role in the progression of prostate cancer. This study confirmed a value of CAMK2N1 in predicting patient's responsiveness, in addition also established a potential of CAMK2N1 as a therapeutic target. Re-expression of CAMK2N1 in prostate cancer may provide clinical benefits to patients; however, further studies are warranted to determine the molecular mechanisms link between CAMK2N1 and androgen receptor signaling in prostate cancer.

## MATERIALS AND METHODS

### Cell culture, Plasmid construction, Reporter genes, Expression vectors, and DNA transfection

Human prostate cancer DU145 and PC3 cells were obtained from ATCC and maintained in DMEM medium (Invitrogen) supplemented with 10% fetal bovine serum. The CAMK2N1 human cDNA clone was purchased from OriGene Technologies and subcloned into the EcoRI/XhoI site of MSCV-IRES-GFP (Addgen) retroviral vector. pGIPZ Vector, pGIPZ-shCAMK2N1(V3LHS-315689, 5'-TCAATAACAACCCGCTTGC-3'), were purchased from Thermo Scientific. DU145 and PC3 cells infected with MSCV-IRES-GFP, MSCV-CAMK2N1-IRES-GFP, pGIPZ-Vector, pGIPZ-shCAMK2N1. GFP positive cells were selected by FACS (Fluorescence Activated cell sorter).

### Colony Formation Assays

Cells were plated in triplicates in 3 ml of 0.3% agarose (sea plaque) in complete growth medium overlaid on 0.5% agarose base, also in complete growth medium. Two weeks post cell seeding, colonies were visualized after staining with 0.04% crystal violet in methanol for 1-2 hrs. The colonies more than 50 μm in diameter were counted using an Omnicon 3600 image analysis system.

### Cell Proliferation Assays

2×10^3^ *stable* cells were seeded in 96-well plate in normal growth medium, and cell growth was measured daily by MTT assays using 3-(4, 5-dimethylthiazol-2-yl)-2, 5-diphenyltetrazolium bromide.

### Cell Cycle and Apoptosis Analysis

Cell cycle parameters were determined by flow cytometry. Stable cells were processed by standard methods using propidium iodide staining of nuclear DNA. Each sample was analyzed by flow cytometry with a FACScan Flow Cytometer (Becton-Dickinson Biosciences, Mansfield, MA) using a 488 nm laser. Histograms were analyzed for cell cycle compartments using ModFit version 2.0 (Verity Software House, Topsham, ME). A minimum of 20,000 events were collected to maximize statistical validity of the compartmental analysis. The PE-Annexin-V Apoptosis Detection Kit (BD Biosciences) was used to detect apoptosis by flow cytometry [19].

### Western Blot

Western blots were performed on DU145, PC3 and LNCaP cells as indicated. Cells were pelleted and lysed in buffer (50 mM HEPES, pH 7.2, 150 mM NaCl, 1 mM EDTA, 1 mM EGTA, 1 mM DTT, 0.1% Tween 20) supplemented with a protease inhibitor cocktail (Roche Diagnostics, Mannheim, Germany). Antibodies used for Western blots were: anti-CAMK2N1 (D-14) [7, 8] and anti-AR (H-280) (Santa Cruz).

### Microarray and Cluster Assays

DNA-free total RNA isolated from DU145 cells expressing CAMK2N1 was used to probe Human 12×135K gene expression array (KangChen Bio-Tech). RNA quality was determined by gel electrophoresis. Probe synthesis and hybridization were performed in NimbleGen Hybridization System. Analysis of the arrays was performed using Agilent GeneSpring GX v11.5.1 software. Arrays were extracted and normalized using NimbleScan v2.5 software, and the *P*-value of 0.05 was applied as a statistical criteria for differentially expressed genes. These genes were then grouped using hierarchical clustering with “complete” agglomeration, and each cluster was further analyzed based upon the known function of the genes contained in the cluster. Expression profiles were displayed using Treeview. Classification and clustering for pathway level analysis were performed by using gene sets ASSESS (Analysis of Sample Set Enrichment Scores), available online. ASSESS provides a measure of enrichment of each gene set in each sample. Gene set enrichment was dependent on a concordance of at least two samples within the replicates that were contrast between phenotypes [20, 21].

### RNA Isolation and Quantitative Real-time PCR (qRT-PCR) Assays

Total RNA was isolated and reversely transcribed to cDNA using TRIzol reagent (Invitrogen) and iScript cDNA Synthesis Kit (Bio-Rad Laboratories, Hercules, CA), respectively, according to the manufacturer's instructions [22]. qRT-PCR was carried out in Bio-Rad CFX96 Real-Time PCR Detection System with iQ SYBR Green Supermix (Bio-Rad). Relative gene expression was normalized to 18s rRNA and calculated by using the 2^-∆∆Ct^ method.

### Immunohistochemistry

Immunohistochemical analysis of human prostate cancer was conducted using a polyclonal CAMK2N1 antibody [7, 8]. Human prostate cancer tissue arrays were purchased from Biomax, US.

### Nude Mice Study

2 × 10^6^ DU145 cells and PC3 cells expressing CAMK2N1 were implanted subcutaneously into 4-6-week-old athymic female nude mice purchased from Beijing HFK Bio-Technology.co., LTD. Tumor growth was measured using a digital caliper every 5 days for 4-5 weeks. Tumor weight was measured when mice were sacrificed on day 32 after cell implantation.

## SUPPLEMENTARY FIGURES


