# Supplementary material for: The tumor suppressive role of CAMK2N1 in castration-resistant prostate cancer
Source: Oncotarget. 2014 May 13;5(11):3611–21. doi: 10.18632/oncotarget.1968 (PMC4116507; doi:10.18632/oncotarget.1968)
Supplement: Supplementary file 1 [file oncotarget-05-3611-s001.pdf]

The tumor suppressive role of CAMK2N1 in castration-resistant prostate cancer

Supplementary Material

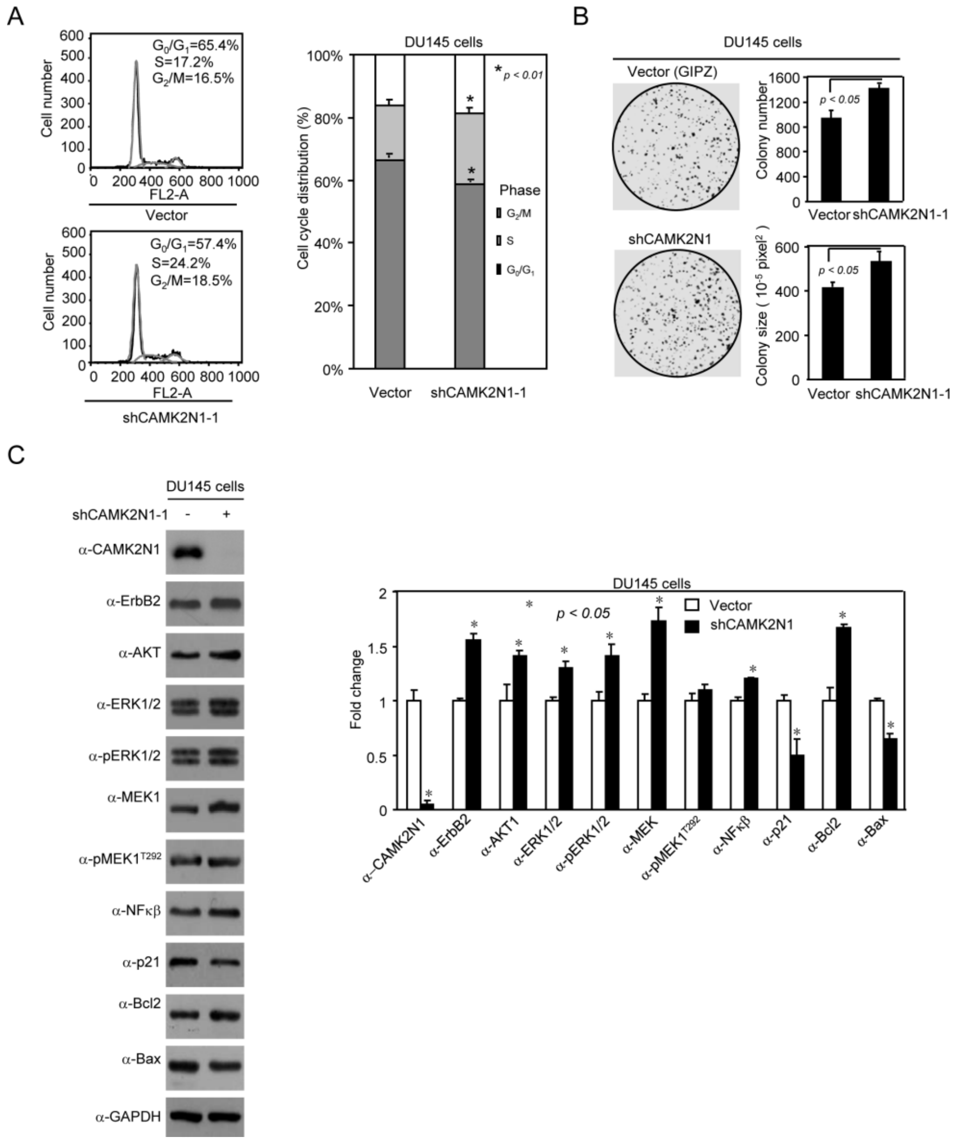

Supplemental Figure 1: CAMK2N1 inhibits cell proliferation, enhances cell-cycle arrest in DU145 cells. DU145 cells with stably knocked down CAMK2N1 were analyzed for (A) cell cycle by FACS, cell proliferation by (B) Colony-forming assay. Expression levels of ErbB2, AKT, MEK1, ERK1/2, NF-κβ, Bcl-2, BAX, and p21 were determined by western blot in DU145 cells (C). Each figure represents three independent experiments.

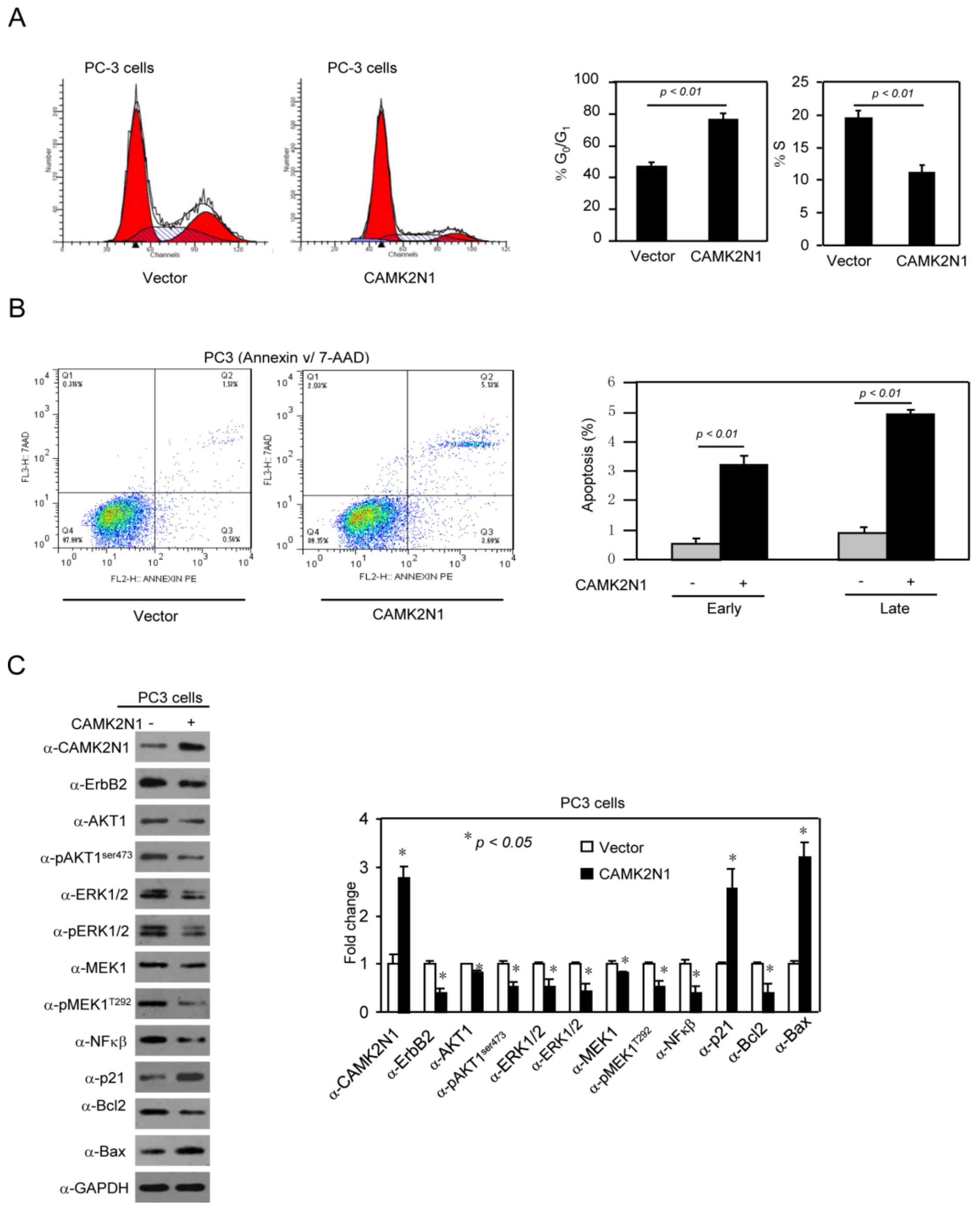

Supplemental Figure 2: CAMK2N1 inhibits cell proliferation and tumor growth, enhances cell-cycle arrest and induces apoptosis in PC3 cells. PC3 cells stably overexpressing CAMK2N1 were analyzed for (A) cell cycle by FACS, (B) apoptosis by Annexin V staining. Quantification of colony numbers and sizes were shown as mean ± SEM for N > 5 separate experiments. (C) Expression levels of ErbB2, AKT, MEK1, ERK1/2, NFκβ, Bcl-2, BAX, and

p21 were determined by western blot in PC3 cells. Each figure represents three independent experiments.

A

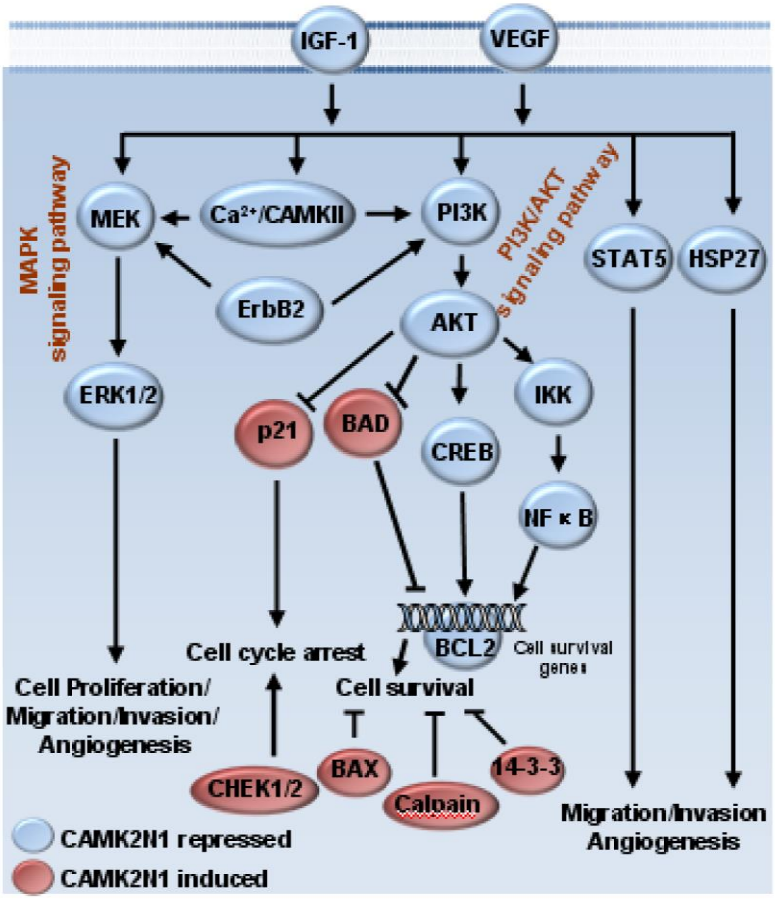

Supplemental Figure 3: KEGG analysis identifies an interconnected network. (A) Analysis includes five major kinase pathways: IGF1, VEGF, ErbB2, MEK/ERK, PI3K/AKT, which are involved in cell growth, survival, migration/invasion and angiogenesis.
